# 

**Published:** 1897-08

**Authors:** 


### THE NORTH CAROLINA VETERINARY MEDICAL ASSOCIATION

Will hold a meeting at Charlotte on August 24, 1897. A generous welcome will be extended to all visiting veterinarians and any who may be sojourning toward Nashville, or through the South, to stop at Charlotte and attend this gathering.

### THE PENNSYLVANIA STATE VETERINARY MEDICAL ASSOCIATION

Will meet on September 21, 1897, at Franklin. They will be welcomed by Mayor George B. Jobson, one of their Vice-Presidents, and accorded the freedom of the city. Franklin is the centre of one of the most thrifty sections of our State. All the adjacent counties have many progressive towns, where are located good, active, earnest members of the profession who should be in the State organization, and the visit of the Association should accomplish much in this direction.

The meeting will be held in one of the best halls in the city, and the attractions adjacent to Franklin are such as to invite a large attendance from the eastern part of the State.

President Rayner in his annual address will present many interesting points of the early history of the veterinary profession in Pennsylvania, of the schools that were early in the field in Philadelphia and Burlington, N. J.

Drs. Leonard Pearson, W. S. McCray, George B. Jobson, Jr., Otto G. Noack, and W. Horace Hoskins have promised to contribute papers for this meeting.

Veterinarians from Ohio and Southwestern New York will be very welcome to attend and take part in the convention's proceedings.

